# *Clostridium difficile* septic arthritis and periprosthetic joint infection in a patient with acute lymphoblastic leukaemia, T-/B-lymphocytopenia and hypogammaglobulinemia – a case report and review of the literature

**DOI:** 10.1099/acmi.0.000233

**Published:** 2021-05-10

**Authors:** Daniel Karczewski, Maximilian Müllner, Carsten Perka, Michael Müller

**Affiliations:** ^1^​ Department of Orthopaedics, Center for Musculoskeletal Surgery, Charité – University Medicine Berlin, Charitéplatz 1, 10117 Berlin, Germany

**Keywords:** septic arthritis, *Clostridium difficile*, periprosthetic joint infection, hypogammaglobulinemia, ALL

## Abstract

To the best of our knowledge, we report the first *Clostridium difficile* infection in a native hip joint with subsequent prosthetic joint infection in a patient at a state of hypogammaglobulinemia. The infection developed following chemotherapy for B-cell precursor acute lymphoblastic leukaemia (BCP-ALL). After chemotherapy, hip arthroplasty was performed for destructive septic arthritis. However, infection in the hip persisted with several failing revisions for more than 3 years, until ultimately hypogammaglobulinemia and T-/B-lymphocytopenia were diagnosed, and supplementation with i.v. immunoglobulins was able to achieve infection control.

## Introduction

*Clostridioides difficile* (family *Peptostreptococcaceae *, genus *Clostridioides *, species *C. difficile *) is a well-studied anaerobic bacterium in the context of nosocomial diarrhoea [1] Despite its well-known intestinal effects, there have been less than ten reported cases of periprosthetic joint infections (PJIs) caused by *C. difficile* to date [2]. Although *C. difficile* infections in native joints are described in sickle-cell-disease patients [3, 4], to the best of our knowledge, we report the first primary hip/acetabulum infection caused by *C. difficile* in a patient at a state of hypogammaglobulinemia, that later persisted as a PJI. The patient developed the initial infection while successfully undergoing chemotherapy for a common B-cell precursor acute lymphoblastic leukaemia (BCP-ALL), later had to undergo total hip arthroplasty (THA) for a destructive septic arthritis, demonstrated *C. difficile* PJI, infection persistence with several failing revisions, and was ultimately diagnosed with hypogammaglobulinemia and T-/B-lymphocytopenia. The entire diagnostic and treatment process went on for almost 5 years, with 13 hip surgeries performed, and an interdisciplinary involvement of 10 different medical subspecialties.

The case report was performed based on a retrospective analysis of our hospital records, personal communication with the involved physicians, as well as a personal patient interview.

Treatment was performed by a separate and specialized interdisciplinary team consisting of orthopaedic surgeons, pathologists and infection specialists, involved in treating patients with musculoskeletal infections such as PJI or septic arthritis. Involved infection specialists are board certified in internal medicine, part of the regular orthopaedic team, and only focus on musculoskeletal infections.

Every microbe detected in our hospital (tissue samples, sonication, joint aspiration, smears) was tested for resistance and classified as sensitive, intermediate or resistant to essential (*n*=10 to 15) antimicrobials. In addition, all antibiotics were started, adjusted and stopped based on the recommendations of our infection specialists, an algorithm-based treatment model, and the individual patient case, including liver/renal function, co-infections, allergies, prior infection history, possible infection focus and secondary diseases/age.

In the course of the entire diagnosis, *C. difficile* was identified in 14 different tissue samples, three aspirations of joint fluid, two times in sonication fluid of removed foreign material and one time via a superficial smear. Cultivation was performed in an anaerobic Schaedler agar for 14 days in cases of tissue and sonication, and for 5 days in the case of a blood culture. Different microbes were identified using a matrix-assisted laser desorption/ionization time of flight (MALDI-TOF) mass spectrometry, while an automatized microdilution procedure (Vitek-System, Biomerieux, Nürtingen, Germany) was used to determine microbe’s resistance to antimicrobials.

## Case report

**Fig. 1.**
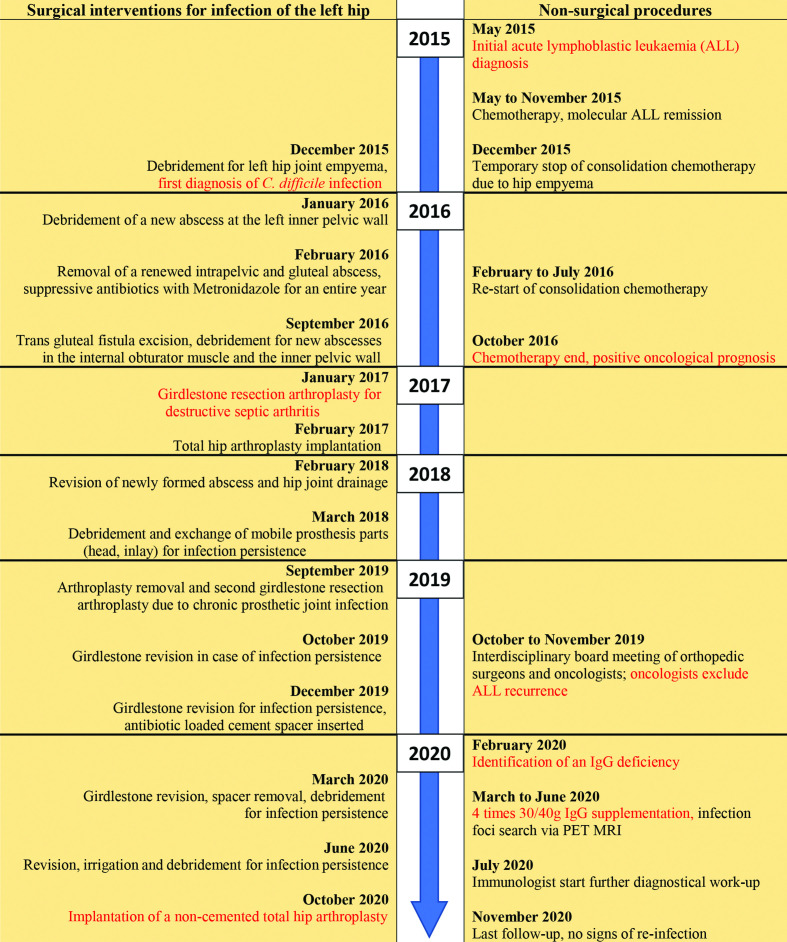
Overview of diagnosis and treatment.

An overview of the entire treatment and diagnostical process is shown in Fig. 1. Key interventions and results are marked in red.

### Beginning of chemotherapy

In May 2015, an otherwise healthy male in his twenties was diagnosed with a BCP-ALL. A therapy based on the GMALL recommendations (German Multicenter Study Group for Adult Acute Lymphoblastic Leukemia) was initiated, adequate ALL remission could be shown, and the patient was released in July 2015. In August 2015, proof of complete molecular remission was detected. In November 2015, signs of inflammation in the blood work-up, as well as increased pain were noted in the left pelvis/hip area. At the beginning of December, the hip showed restricted movement and severe pain. *C. difficile* was isolated via CT-guided hip puncture, an arthroscopic debridement was performed 3 days later, and therapy with piperacillin/tazobactam and Ciprofloxacin was started.

### End of chemotherapy

In January 2016 the patient re-presented with increasing infection parameters. CT imaging showed a pelvic abscess, that was successfully drained. No pathogen detection could be obtained from abscess tissue/fluid (pus) (two tissue samples were sent in for cultivation). After both clinical signs of infection (local swelling, pain, fever) and C-reactive protein (CRP) were in regression, the i.v. antibiotic therapy could be stopped, the patient was discharged, and the antibiotics adapted to a prolonged oral therapy (metronidazole and rifampicin according to microbial resistance). In February 2016, a CT scan was performed due to renewed hip pain, and showed an abscess under the iliacus and piriformis muscles. A further debridement was able to consolidate the hip. Following the adjustment of the antibiotics, and a decrease of the infection parameters, the patient was successfully discharged, and the oral antibiotics with metronidazole were continued for a total of 1 year (3×400 mg per day). From February to July 2016, the patient noted no symptoms of infection or pain. However, in the following months pain in the left hip increased gradually and, despite continued suppressive therapy with metronidazole, a new trans gluteal fistula developed. At the beginning of September 2016, a further debridement for abscess formation was performed, and the trans gluteal fistula was excised. *C. difficile* and *Staphylococcus hominis* were identified posteriorly from samples taken during surgery. During the stay, no gastrointestinal colonization with *C. difficile* could be confirmed. In October, the haematology-oncology department finally evaluated the patient. Given a standard risk ALL with a completed therapy protocol and a young patient age, a positive long-term prognosis was given.

### Total hip arthroplasty

In January 2017, a CT presented a chronic destructive septic arthritis of the left hip and osteomyelitis of the acetabulum extending into the left ilium. Given the grade of joint destruction, a girdlestone resection was performed. Identification of *C. difficile* was possible after cultivation of three (out of four) intraoperatively obtained tissue samples, and the postoperative calculated antimicrobial therapy was accordingly adjusted. The patient was discharged without clinical signs of infection, and readmitted 6 weeks later for a cementless THA. A *C. difficile* positive tissue sample was obtained during the surgery. Following the patient discharge, metronidazole was prescribed orally for 1 year. In February 2018, the patient re-presented with new signs of infection, surgical abscess drainage was performed, repeated 9 days later, and obtained abscess drainage fluid was cultured without subsequent detection of a microbe. Since no fistula was identified between the abscess and the hip joint, only mobile parts of the prosthesis were exchanged. Metronidazole was prescribed for a total of 3 years as a long-term suppressive therapy (3×500 mg per day).

### Second girdlestone resection

In September 2019, MRI results demonstrated osteomyelitis around the THA spreading to the acetabulum. The THA was removed, a second girdlestone situation created, and the iliac cavity irrigated. *C. difficile* was identified as the causing microbe. Despite adequate antibiotics according to the microbial susceptibility and complete prosthesis removal, the patient presented with newly formed fistulas, so that a second look debridement was performed in October (identification of *C. difficile *), and later in December 2019 an antibiotic loader spacer was implanted to reduce anatomic death space (no microbe identification). In October/November 2019, the oncologists excluded ALL recurrence.

### Hypogammaglobulinemia

In February 2020, the haematologists performed a work-up of the patient’s immunology response, and identified an antibody deficiency with a partial IgG subclass deficiency, with measured IgG values of 4.8 to 5.5 g l^−1^ (physiological range 7–16 g l^−1^). An i.v. immunoglobulin replacement (IVIG) therapy was started in March, with four doses of 30 or 40 g IVIG administered until June. At the end of June, the last identified IgG level was at 5.9 g l^−1^. In March 2020 the patient presented with signs of a persistent infection, that subsequently led to a renewed girdlestone revision, a spacer removal and debridement. *Staphylococcus epidermidis* and unspecified *Clostridioides* were identified postoperatively out of intraoperatively obtained samples. A whole-body PET-MRI was performed in May 2020, and excluded a possible yet undetermined infection focus. Despite aggressive antibiotic regimen, newly started IVIG therapy and removal of all foreign material in the hip, the patient returned with signs of reinfection only 7 weeks after the last revision, at the beginning of June. Like in prior revisions, debridement was performed, antibiotics adjusted and *C. difficile* identified after cultivation of intraoperatively obtained samples.

### THA reimplantation

In July 2020, the immunology department began a complete work-up of the patient’s blood values (146 parameters), and a focused anamnesis was performed. Before 2015, there was no increased tendency of infection or other serious diseases. The entire family anamnesis was negative for any kind of primary immunodeficiency. The blood work-up revealed: (1) a mild T-lymphocytopenia (including native CD4, mature CD4 and CD8 subtypes), (2) reduced class switched memory B-cells, (3) and increased transitory B-cells. The immunoglobins did not show significant abnormalities given their recent supplementation. In October 2020, after more than a year without a hip joint, THA reimplantation was performed, with IVIG supplementation 3 weeks directly prior to the operation. At the latest follow-up in November, no signs of reinfection were noted.

## Discussion

To the best of the authors' knowledge, this is the first case of destructive septic arthritis caused by *C. difficile* in a native hip joint in a patient at a state of hypogammaglobulinemia. Neither described is any case of *C. difficile* associated PJI in a patient with hypogammaglobulinemia and T-/B- lymphocytopenia following an ALL treatment. Only 1 % of all infections with *C. difficile* occur extra intestinally, with reported infection sites including peritonitis, reactive arthritis, fistulae, abdominal and brain abscess [5, 6]. While this is not the first case of *C. difficile* in a native hip joint [3, 4], to the best of the authors' knowledge, it is the first case in a patient at a stage of hypogammaglobulinemia. Rare cases of *C. difficile* associated osteomyelitis and septic arthritis have been reported in the context of sickle-cell anaemia [3, 4, 7], and PJI, oftentimes associated with immunosuppression [2]. The exact role of *C. difficile *-associated diarrhoea in the pathogenesis of extra intestinally infections remains unknown. Our patient did not suffer from *C. difficile *-associated diarrhoea at any point. In this context, haematogenous and local routes should be examined as theoretical ways for primary infection in the future.

Prior reports of *C. difficile *-associated PJI emphasize the importance of adequate wound revision in order to control the infection of the prosthesis and the surrounding tissue [2]. The main problem in this case included the persistence of infection despite adequate antibiotics according to the microbial susceptibility and resistance patterns, even during a girdlestone situation. Up to this point, it remains unknown if the hypogammaglobulinemia and the T-/B-lymphocytopenia existed before the onset of the ALL, if the chemotherapy was the causing initial event, or if the IVIG therapy will be able to control the infection in the long run.

A study by Duraisingham *et al*. analysed 167 patients with primary or secondary antibody deficiencies that received immunoglobulin supplementation treatment [8]. As in the present study, the authors were able to show significant infection reduction in both primary and secondary antibody-deficient patients following immunoglobulin supplementation. Although our immunology department was not able to identify if our patient´s hypogammaglobulinemia and T-/B-lymphocytopenia was of primary or secondary origin, risk factors for a secondary hypogammaglobulinemia described by Duraisingham *et al*., such as Rituximab and chemotherapy, were also identified in the present case [8]. However, if on the contrary the patient’s immunodeficiency was of primary origin, it could also have remained silent until chemotherapy was performed for ALL. In this context, a prospective cohort study by Ameratunga *et al*. analysing Common Variable Immunodeficiency Disorders (CVIDs) was able to identify a total of 120 patients with untreated primary hypogammaglobulinemia, of whom 59 were asymptomatic. Patients with moderate hypogammaglobulinemia (defined as IgG 3.0–6.9 g l^−1^ by the authors) were healthy for a mean of 8 years, which in return corresponds well to the measured results identified in our patient (4.8–5.5 g l^−1^), especially when assuming a possible primary origin of deficiency [9].

A descriptive study by Diaz-Ledezma *et al*. points out that there might be a possibility that hypogammaglobulinemia could be another risk factor for PJIs [10]. Driessen *et al*. suggested adapting the IVIG dosage based on an individual decision influenced by the grade of IgG reduction and the susceptibility to infections [11]. Referring to the German guideline for therapy of immunodeficiencies, an initial dose of at least 400 mg per kilogram body weight per month is recommended and the lowest level should not fall below 4.5 g l^−1^ [12]. Due to the limited status of studies, there is no information concerning the time interval between the administration and the exact dose, so both parameters must be selected individually in cases of musculoskeletal infection.

Hypogammaglobulinemia in the context of PJI has rarely been described (Table 1) [13–16]. All cases that we were able to identify were associated with a type of lymphoma. In contrast to our procedure, no previous study has described a treatment protocol involving IVIG replacement therapy, instead, a surgical and/or additional antimicrobial therapy was the primary treatment of choice in prior studies. Similar to *C. difficile* in this article, rare microbes such as *Mycoplasma salivarium *, *Ureaplasma urealyticum* or *Campylobacter jejuni* were identified. Given short follow-ups and heterogenous outcome characteristics, a final evaluation of existing cases is challenging. The largest study of Abad *et al*. reported that 5 out of 6 patients died during the follow-up [13], while Roerdink *et al*. reported of an infection free and mobile patient [15].

**Table 1.** Hypogammaglobulinemia and PJI

Study	Demographics	Microbe	Underlying disease	Treatment strategy	Follow-up and outcome
Abad *et al.* (2019) [13]	Median age of 65.5, three hips, two knees, one elbow	(3) Coagulase negative staphylococci, (2) *Staphylococcus aureus * (1) *Streptococcus pneumoniae *	All with lymphoid malignancy and a haematopoietic stem cell transplantation	i.v. antimicrobial therapy and surgery in all patients	Last follow up: five out of six patients were deceased
Thoendel *et al.* (2017) [14]	Right knee, 52, male	* Mycoplasma salivarium *	CVID with hypogammaglobulinemia Follicular B-cell lymphoma in remission Seronegative inflammatory arthritis	Two-stage exchange Antibiotic-loaded nonarticulating spacer 6 weeks of intravenous vancomycin and cefepime	9 months following reimplantation; constant sinus tract with Mycoplasma salivarium under therapy with doxycycline
Roerdink *et al*. (2016) [15]	Bilateral knees, 69, female	* Ureaplasma urealyticum *	Non-Hodgkin lymphoma (under remission treated with R-CHOP scheme)	Arthroscopic debridement and lavage Moxifloxacin 400 mg once a day and doxycyclin 100 mg twice a day Bilateral exchange of mobile parts	6 months follow up; knee functionality on both sites intact
Diaz-Ledezma *et al.* (2014) [10]	Right knee, 78, male	n.a.	n.a.	n.a.	n.a.
Peterson *et al*. (1993) [16]	Left hip, 60, male	* Campylobacter jejuni *	B-cell lymphoma Haemophilia A AIDS Hepatitis B cirrhosis	i.v. gentamicin (later erythromycin) and ciprofloxacin	Patient did not relapse through September 1992

Genetic analysis is a possible option to determine an underlying defect in selected patients. Given the complexity and costs associated with genetic counselling, selection of patients must be based on various warning signs, such as opportunistic infections, protracted course or degree of severity of infection, and malignant diseases such as B-cell lymphomas. In this context, neutropenia, lymphocytopenia and thrombocytopenia can be side effects of an uncomplicated viral infection or chemotherapy, but can also be possible first indications of an underlying immunodeficiency. If a primary immune defect is suspected, a blood count with differentiation of the immunoglobulins is recommended [10, 17, 18].

During the entire treatment and diagnostical period, all samples identified, demonstrated *C. difficile* sensitivity to metronidazole. In addition, vancomycin was used as an additional treatment in patients, especially as an i.v. option. Although *C. difficile* resistance against classical antibiotics (vancomycin, metronidazole) was not identified in the present study, long-term antibiotic therapy bears the risk of bacterial resistance formation, especially in recurrent infections. In this context, non-traditional treatment options such as nitazoxanide, teicoplanin and fidaxomicin may be considered alternative options for *C. difficile* reinfections [19]. According to Cocanour *et al*. alternatives to metronidazole and vancomycin can be considered following a third recurrence of infection [20]. However, limited experiences with these alternative agents in systemic and potentially life-threatening *C. difficile* infections are present, as most were evaluated in the context of gastrointestinal infections [21].

## Conclusion

A PJI caused by *C. difficile* is a rare entity. To the best of the authors' knowledge, we report the first case of *C. difficile *-associated native arthritis with subsequent development of a PJI in a patient with hypogammaglobulinemia and lymphocytopenia during and following an ALL, but without a sickle-cell disease. IVIG treatment following the hypogammaglobulinemia demonstrated promising early results.
